# Effects of Autonomous Sensory Meridian Response on the Functional Connectivity as Measured by Functional Magnetic Resonance Imaging

**DOI:** 10.3389/fnbeh.2020.00154

**Published:** 2020-08-27

**Authors:** Seonjin Lee, Jooyeon Kim, Sungho Tak

**Affiliations:** ^1^Research Center for Bioconvergence Analysis, Korea Basic Science Institute, Cheongju, South Korea; ^2^Graduate School of Analytical Science and Technology, Chungnam National University, Daejeon, South Korea; ^3^Center for Research Equipment, Korea Basic Science Institute, Cheongju, South Korea

**Keywords:** autonomous sensory meridian response, functional connectivity, functional magnetic resonance imaging, default mode network, affective touch network, self-network

## Abstract

Autonomous sensory meridian response (ASMR) is a sensory phenomenon in which audio-visual stimuli evoke a tingling sensation and is accompanied by a feeling of calm and relaxation. Therefore, there has been an increasing interest in using stimuli that elicit ASMR in cognitive and clinical neuroscience studies. However, neurophysiological basis of sensory-emotional experiences evoked by ASMR remain largely unexplored. In this study, we investigated how functional connectivity is changed while watching ASMR video, compared to resting state, and assessed its potential association with affective state induced by ASMR. 28 subjects participated in fMRI experiment consisting of 2 sessions (resting-state and task of viewing ASMR-eliciting video). Using a seed-based correlation analysis, we found that functional connections between the posterior cingulate cortex, and superior/middle temporal gyri, cuneus, and lingual gyrus were significantly increased during ASMR compared to resting state. In addition, we found that with the pregenual anterior cingulate cortex seed region, functional connectivity of the medial prefrontal cortex was increased during ASMR condition, relative to resting state. These results imply that ASMR can be elicited and maintained by ongoing interaction between regional activity that are mainly involved in the mentalizing and self-referential processing. We also found that ASMR-induced affective state changes (high activation negative and high activation positive state) were negatively correlated with functional connectivity involved in visual information processing, suggesting that visual information processing in response to high arousal states can be weakened by ASMR-eliciting stimuli.

## Introduction

Stress is common in everyday life, and is believed to affect individual health and happiness (Segerstrom and Miller, 2004; Cohen et al., 2007). As a result, the development of stress management approaches has become an important endeavor of preventing stress-related health problems and accomplishing psychological well-being. In recent years, the autonomous sensory meridian response (ASMR) videos have been widely used in the management of stress, by inducing relaxation and sleep (Barratt and Davis, 2015; Lee et al., 2019). Specifically, ASMR is a sensory phenomenon in which individuals experience a tingling in the head and neck, in response to specific triggering audio and visual stimuli (Barratt and Davis, 2015). The ASMR triggers lead to response of psychologically pleasant effects such as feeling of relaxation, reduction in anxiety, and sleep induction (Barratt et al., 2017; Cash et al., 2018; Poerio et al., 2018).

Several studies have explored the neurophysiological basis of ASMR using functional magnetic resonance imaging (fMRI) (Smith et al., 2017, 2019; Lochte et al., 2018). Specifically, Lochte et al. (2018) examined the brain activation during ASMR, and observed significant activation in regions of the medial prefrontal cortex (mPFC), dorsal anterior cingulate cortex, supplementary motor area, and insular cortex during ASMR condition, compared to the brain activity during resting state.

Smith et al. (2017, 2019) investigated the differences of resting-state network between ASMR experienced and non-ASMR experienced individuals. Using an independent component analysis (Beckmann et al., 2005), they found that participants with ASMR had less connections of the precuneus with other regions of the default mode network (DMN) than controls. These previous studies demonstrated the associations of ASMR with the changes in regional activity and networks of resting state. However, it is still unclear how connections among brain regions are explicitly modulated by ASMR.

To address this issue, this paper focuses on the investigation of ASMR condition-specific functional connectivity changes in a brain network, compared to the resting-state functional connectivity, using 3T functional magnetic resonance imaging (fMRI). Functional connectivity was assessed using a seed-based correlation approach (Biswal et al., 1995; Whitfield-Gabrieli and Nieto-Castanon, 2012). We hypothesized that ASMR condition would change the functional connectivity within the brain network involved in mentalization and self-referential processing as a meditation effect of ASMR. This is based on a previous study (Barratt and Davis, 2015) reporting that sitting quietly while watching relaxed scenes to arouse ASMR for a certain period of time could be regarded as a form of mindfulness. Mindfulness meditation can arouse relaxed and calm states by developing a level of mentalization that controls emotion using a capacity for resilience in the face of distressed conditions (Sharp et al., 2011; Bateman and Fonagy, 2013). Also, the meditation has been known to induce positive emotion using self- and other-referential processing (Logie and Frewen, 2015). The previous study (Logie and Frewen, 2015) has shown that participants who experienced mindfulness meditation had self-positive bias that led to positively affective responses during experimental self- and other-referential processing. Therefore, based on an association of ASMR and meditation conditions, we tested our hypothesis by investigating the ASMR condition-specific connectivity changes in the DMN that are involved in the mentalizing (Lombardo et al., 2010; Mars et al., 2012), and the self- and other-networks that are associated with self- and other-referential processing (Northoff et al., 2006; Murray et al., 2015). The self-network has a function of self-specific processing, indicating non-self-/self-distinction to comprehend self in domain of perception, emotion, and cognition (Northoff et al., 2006). The other-network has a function of other-specific processing that represents other-/self-distinction in understanding others’ mental and emotional states across the domains of perception, emotion, and cognition (Murray et al., 2015).

In addition, since the ASMR triggers have been known to induce a tingling sensation as a secondary phenomenon resulting from intensely positive emotion (Barratt and Davis, 2015), we explored the changes in the functional connectivity of the affective touch network while watching the ASMR stimuli (Morrison, 2016). We selected the seed regions for the default mode, affective touch, and self-/other-networks as follows. The posterior cingulate cortex (PCC), mPFC, and left/right lateral parietal cortex (lLPC, rLPC) were used as the seed regions for the DMN, because these regions are recognized as central hubs within the network (Greicius et al., 2003). For the affective touch network, we used the right posterior insular cortex (Ig2) as a seed region based on a previous meta-analysis study (Morrison, 2016). Morrison (2016) reported a higher activation of Ig2 in response to affective touch compared with discriminative touch. Using this seed region of Ig2, they observed an affective touch network composed of bilateral clusters, including posterior and anterior insular cortex, postcentral primary, and secondary somatosensory regions. For the self- and other-networks, we used the pregenual anterior cingulate cortex (pACC) and posterior cingulate cortex/precuneus (PCC/PC) regions as seed ROIs, because these two seed regions have been reliably shown to be involved in conceptual self- and conceptual other-processing, respectively (Murray et al., 2012). The self-network consisted of the pACC and anterior insular cortex, whereas the other-network consisted of the PCC/PC and angular gyrus/temporoparietal junction (Murray et al., 2015).

Finally, using the functional connectivity estimates, we further investigated the potential association of condition-specific connectivity changes with affective state changes while watching ASMR stimuli. Our hypothesis was that the changes in functional connectivity during ASMR would be closely associated with the changes in pleasant/unpleasant emotion and arousal states during ASMR. We assessed the affective outcomes of watching ASMR video clips using the Multi-Affect Indicator (Warr, 1990; Warr et al., 2014) and then performed a correlation analysis between the functional connectivity strengths and individual scores for affective state induced by ASMR.

## Materials and Methods

### Participants and Experimental Protocol

Twenty-eight healthy subjects (13 females, 15 males; mean age: 26.39 ± 3.77 years) participated in this study. No subjects had any history of neurological disorders. The study was approved by the Institutional Review Board (IRB) of Korea Basic Science Institute, and the experiment was performed with the understanding and written consent of each participant, according to IRB guidelines.

The experiment consisted of two sessions. In the first session, which served as a control experiment, participants underwent a 5-min resting-state fMRI scan. During this scan, participants were instructed to stare at a fixation point in the center of the screen and remain awake. The scan duration of 5 min was based on previous studies showing that estimates of resting-state functional connectivity stabilized with this acquisition time (Van Dijk et al., 2010). We also determined the specific instructions for resting-state condition (eyes closed, eyes open, or eyes fixated on a cross), based on Patriat et al. (2013). It was found that reliability in the default mode, attention, and auditory networks was the highest when subjects kept their eyes fixated on a cross.

In the second session, participants underwent ASMR task in the MRI scanner. During the scan, participants were instructed to view ASMR-eliciting video for 5 min. This video was trimmed to a length of 5 min from the full-length version of the YouTube video, which comprised repetitive and slow movements with a scratching sound (i.e., scratching of a sand table). The web address is as follows: https://youtu.be/bCFALoEfBGw. While standards for ASMR videos have not yet been extensively examined, several studies (Barratt and Davis, 2015; Fredborg et al., 2017) have established the common stimuli that elicit an intense ASMR experience, including whispering, scratching sound, and slow/repetitive movements. Therefore, we selected the content of the video clips based on these criteria. The length of ASMR video clips was set to be consistent with that of the resting-state condition because the scan length has been known to affect the reliability of fMRI connectivity estimates (Birn et al., 2013).

After completing fMRI experiments, outside the scanner, participants responded to questionnaires for assessing the changes in affective states while watching ASMR video clips (see the Behavior Data Analysis section for more details). Overall, this study consisted of three phases: the first session for resting-state experiment in the MRI scanner (5 min), the second session for ASMR experiment in the MRI scanner (5 min), and behavioral data collection outside the scanner.

### MRI Acquisition

All images were acquired using a 3T Philips Achieva scanner (Philips Medical Systems, Best, The Netherlands). Structural images were acquired using a three-dimensional T1-weighted sequence [repetition time (TR) = 6.6 ms; echo time (TE) = 3.1 ms; flip angle = 9°; voxel size = 1.0 × 1.0 × 1.2 mm^3^; field of view (FOV) = 240 mm; 170 slices]. Blood oxygenation level dependent (BOLD) images were obtained using a T2^∗^-weighted gradient echo-planar imaging (EPI) sequence (TR = 2000 ms; TE = 35 ms; flip angle = 79°; voxel size = 3.0 × 3.0 × 3.0 mm^3^, FOV = 195 mm, 34 interleaved slices without slice gap).

### Data Processing

The functional connectivity toolbox (CONN toolbox, Whitfield-Gabrieli and Nieto-Castanon, 2012) with the statistical parametric mapping software package (SPM12, Friston et al., 2007) was used for pre-processing of the functional and structural images, and functional connectivity analysis.

The effects of head movement between scans were corrected by realigning all scans to the first image using a six-parameter affine spatial transformation; the geometric distortion was corrected by the unwarp function. The ensuing realignment parameters were saved for modeling residual head motion effects in the BOLD time series. To further mitigate motion-related BOLD effects, including spikes, we used artifact detection tools (ART, https://www.nitrc.org/projects/artifact_detect) interoperable with CONN toolbox. Specifically, outlying volumes in BOLD time series (scan “scrubbing”) were identified based on normalized global mean intensity values (>Z = 5) and motion parameters (>1 mm translational movement in the x, y, or z planes or >0.02 rotation in yaw, pitch, or roll). The matrices of outliers and realignment parameters were then entered as first-level covariates (i.e., nuisance variables). To compensate for slice-acquisition delays, the signal in each slice was realigned temporally to a reference middle slice using sinc interpolation. The structural image was co-registered with functional images and segmented into gray matter (GM), white matter (WM), and cerebrospinal fluid (CSF). All images were spatially normalized to the Montreal Neurological Institute (MNI) space. Spatial smoothing with a 6 mm full-width at half-maximum (FWHM) Gaussian kernel was applied to the normalized images.

Systemic physiological confounds arising from cardiac and respiration have been known to cause spurious correlation structures throughout the brain (Birn et al., 2006; Chang and Glover, 2009; Murphy et al., 2013). We therefore reduced systemic physiological noise using the anatomical component-based noise correction method (aCompCor) (Behzadi et al., 2007). The method has also been shown to be effective in the suppression of motion-related artifacts (Muschelli et al., 2014). Assuming that the physiological noise contribution is globally distributed, and neuronal activity-related signals are low in the WM and CSF, the signals within the WM and CSF were used as sources that primarily reflect physiological noise. The top three components obtained from each of the WM and CSF using principal component analysis were included as the nuisance regressors in the first-level analysis. In addition, to remove spurious task-induced co-activation effects, we constructed a condition-specific regressor and included it as additional temporal confounding factors by convolving a canonical hemodynamic response function with a condition (either ASMR or resting-state) spanning the entire scanner acquisition length (Fair et al., 2007; Whitfield-Gabrieli and Nieto-Castanon, 2012). Prior to the first-level connectivity analysis, these temporal confounding factors (consisting of subject movement, cardiac/respiration, and spurious parameters related to task effects) were regressed out from BOLD time series at each voxel. The resulting residual time series were then band-pass filtered in the range of 0.01–0.1 Hz to constrain the low-frequency BOLD fluctuations presumed to be related to spontaneous neural activity (Biswal et al., 1995; Leopold et al., 2003).

First-level functional connectivity maps were generated by computing Pearson’s correlation coefficients between average BOLD time series calculated across all the voxels of a given seed region and the time series of all other voxels in the brain (Biswal et al., 1995; Fox et al., 2005). The resulting correlation coefficients were converted to *Z*-scores using Fisher transformation (Fisher, 1915) to improve the normality assumptions of the subsequent second-level general linear model (GLM) analysis. Functional connectivity considered in our analysis was associated with (a) the DMN (Greicius et al., 2003), (b) affective touch network (Morrison, 2016), and (c) the self-/other-networks (Northoff et al., 2006; Murray et al., 2015). As seeds of the DMN, we used the PCC centered at MNI coordinates [1, −61, 38], mPFC (MNI: [1 55 −3]), and l/rLPC (lLPC, MNI: [−55 −12 29], rLPC, MNI: [56 −10 29]). The seed regions of interest (ROIs) were defined using a standardized CONN toolbox atlas (networks.nii) that was originally derived from group-level independent component analysis (ICA) of the human connectome project dataset (Calhoun et al., 2001; Whitfield-Gabrieli and Nieto-Castanon, 2012; Van Essen et al., 2013). For an affective touch network, we used the Ig2 as a seed ROI that comprised all voxels within a sphere of 6 mm radius, centered on the MNI coordinates [42, −14, 8]. Finally, for the self- and other-networks, we used the pACC and PCC/PC regions as seed ROIs (spheres of 6 mm radius, centered on MNI coordinates: [−2, 38, 16] and [2, −61, 26]).

Following the computation for the first-level functional connectivity maps, the resulting voxel-specific *Z*-scores between a seed area and every other voxel for each subject were entered into a second-level GLM analysis. Specifically, we performed a one-sample *t*-test at the second level to test the statistical significance of each functional connectivity map in a group of subjects that was generated during resting-state or ASMR conditions (ASMR). We then tested our hypothesis that functional connectivity related to mentalizing and self-referential processing within the default mode, affective touch, and self-/other-networks would be greater during an ASMR condition than the resting-state, using a two-tailed paired sample *t*-test with a contrast “ASMR > resting-state” at the second-level. This analysis enabled us to compare the functional connectivity patterns between two conditions, including a resting-state and an ASMR condition, and assess their statistical significance in a sample. For false positive control in the whole-brain seed-to-voxel connectivity analysis, we applied a cluster-forming threshold using a height threshold of uncorrected *p*-value < 0.001 and a cluster-extent threshold of false discovery rate (FDR)-corrected *p*-value < 0.05 (Friston et al., 1994; Whitfield-Gabrieli and Nieto-Castanon, 2012). We used a semi-automated search for finding local maxima (peaks) and their MNI coordinates within the cluster-corrected thresholded map, to identify regions within the significant functional connectivity maps. Their anatomical labels were determined using xjView toolbox (https://www.alivelearn.net/xjview), and the Brodmann area labels were identified using the Brodmann atlas, which is included in the MRIcron software (https://www.nitrc.org/projects/mricron). Functional connectivity maps were overlaid on a cortical surface atlas using the CONN toolbox (Whitfield-Gabrieli and Nieto-Castanon, 2012).

### Behavioral Data Analysis

To investigate the potential association of functional connectivity estimates with the psychological changes of ASMR, we measured the affective outcomes of watching ASMR video clips using the Multi-Affect Indicator (Warr, 1990; Warr et al., 2014). This multi-affect indicator has been designed to specify different kinds of feelings in terms of two dimensions, including the conventional negative-to-positive continuum (from unpleasant to pleasant state) and low-to-high mental activation (arousal) that defines one’s state of readiness for action or energy expenditure (Russell, 2003). Particular feelings were then categorized into four affective states: low-activation positive (LAP, which corresponds to comfort and calmness), high-activation positive (HAP, related to enthusiasm and excitement), low-activation negative (LAN, related to depression and sadness), and high-activation negative states (HAN, related to anxiety and stress). In this study, we used 12 items to measure these affective states (Warr, 1990; Poerio et al., 2018): “calm,” “relaxed,” and “at ease” for LAP; “enthusiastic,” “joyful,” and “excited” for HAP; “depressed,” “dejected,” and “hopeless” for LAN; and “anxious,” “nervous,” and “tense” for HAN. After completing the fMRI experiments, the participants were asked to rate each item in the range of 1 (much less) to 7 (much more) by responding to the question: How did you feel while watching ASMR video clip during the MRI scan, compared to before you watched the video?

We then performed two-tailed paired samples *t*-tests to compare the means of two affective states that were selected from LAP, HAP, LAN, and HAN, and determined whether there was a significant difference between the two states that can be observed from ASMR stimuli. In addition, we performed a correlation analysis to investigate the associations of these affective state changes with ASMR condition-specific functional connectivity changes. Specifically, for each brain network, we identified clusters that had a significantly higher functional connectivity from a seed region for ASMR condition than the resting-state condition (a height threshold of uncorrected *p*-value < 0.001 and a cluster-extent threshold of FDR-corrected *p*-value < 0.05). Then, we extracted the functional connectivity values (z-score) of peak coordinates (i.e., the local maxima of the cluster) for all subjects, and calculated Pearson’s correlation coefficients between these functional connectivity strengths and individual scores for each affective state. We decided that the computed correlation value is significantly different from zero if the *p*-value is less than 0.05.

## Results

### Functional Connectivity

**FIGURE 1:**
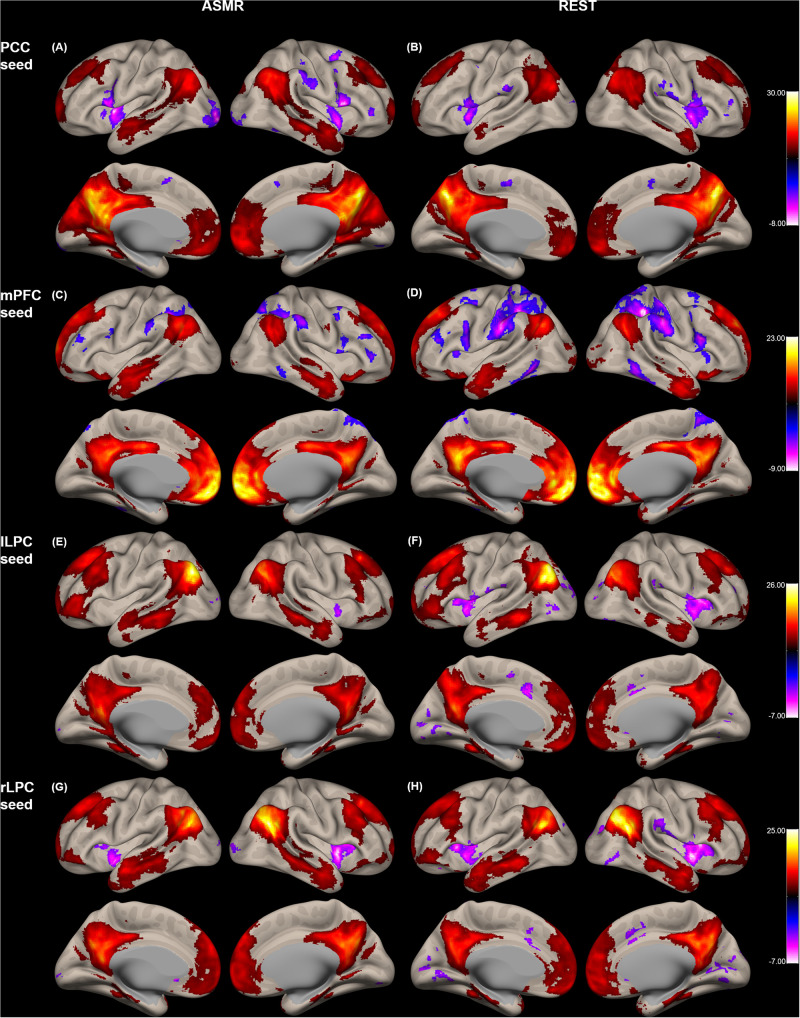
Group-level functional connectivity of the *t*-statistic in the default mode network during resting-state, and in response to ASMR effects. Functional connectivity strengths in terms of *t*-statistics were thresholded at a significance level of false discovery rate (FDR)-corrected *p* < 0.05, and overlaid on a cortical surface atlas. Functional connectivity of the posterior cingulate cortex (PCC) seed region in response to ASMR **(A)**, and in resting-state **(B)**. Functional connectivity of the medial prefrontal cortex (mPFC) seed region in response to ASMR **(C)**, and in resting-state **(D)**. Functional connectivity of the left lateral parietal cortex (lLPC) seed region in response to ASMR **(E)**, and in resting-state **(F)**. Functional connectivity of the right lateral parietal cortex (rLPC) seed region in response to ASMR **(G)**, and in resting-state **(H)**.

Figure 1 shows the group-level functional connectivity of the *t*-statistic in the default mode network generated during either ASMR or resting-state conditions. Statistical significance of clusters and their peak coordinates for ASMR and resting-state conditions are summarized in Tables 1, 2, respectively. While the global maxima of the functional connectivity was located in the seed cluster, in both conditions of resting-state and ASMR, the significant hubs (local maxima of the functional connectivity within the cluster) were reliably positioned in the PCC, mPFC, lLPC, rLPC, and superior/middle/inferior temporal gyri, and superior/inferior frontal gyri. For seed regions of the PCC and rLPC, the negative functional connectivity was observed in the insular cortex.

**TABLE 1:** Statistical significance of the group-level functional connectivity generated during ASMR condition.

Connectivity (ASMR)	Brodmann area	MNI (x,y,z)	*Size*	*Peak-T*	*Peak-beta*	*Size p-FDR*
**PCC seed**						
Precuneus	BA 7	(−2, −64, 40)	25801	30.715	0.977	0.00000
Medial frontal gyrus	BA 10	(4, 50, −6)	12173	11.445	0.342	0.00000
Angular gyrus	BA 39	(54, −62, 34)	6167	14.407	0.436	0.00000
Insular cortex	BA 48	(−36, 4, 2)	1195	−8.276	−0.170	0.00000
Cuneus	BA 18	(−26, −100, −8)	631	−7.666	−0.169	0.00000
Supramarginal gyrus	BA 1	(64, −24, 48)	355	−5.303	−0.204	0.00000
Cuneus	BA 17	(20, −102, −4)	221	−5.441	−0.144	0.00000
Superior frontal gyrus	BA 8	(22, 4, 54)	151	−5.831	−0.135	0.00004
**mPFC seed**						
Medial frontal gyrus	BA 10	(2, 60, −2)	22584	29.669	1.241	0.00000
Posterior cingulate cortex	BA 23	(6, −50, 22)	6927	14.525	0.562	0.00000
Angular gyrus	BA 39	(−50, −66, 32)	2558	11.710	0.487	0.00000
Postcentral gyrus	BA 40	(54, −32, 40)	1832	−6.744	−0.229	0.00000
Superior temporal gyrus	BA 21	(60, −58, 20)	1715	10.946	0.397	0.00000
Inferior temporal gyrus	BA 20	(54, −4, −36)	1659	8.214	0.256	0.00000
Inferior frontal gyrus	BA 45	(−44, 38, 16)	251	−5.211	−0.241	0.00000
Superior temporal gyrus	BA 38	(36, 20, −36)	146	6.139	0.163	0.00006
Parahippocampal gyrus	BA 30	(26, −32, −16)	144	5.305	0.132	0.00006
Inferior frontal gyrus	BA 45	(46, 38, 4)	109	−5.598	−0.212	0.00046
**lLPC seed**						
Superior fontal gyrus	BA 8	(24, 32, 48)	16420	14.658	0.390	0.00000
Angular gyrus	BA 39	(−44, −72, 32)	15617	25.964	1.003	0.00000
Angular gyrus	BA 39	(46, −70, 36)	5690	18.819	0.645	0.00000
Fusiform gyrus	BA 37	(36, −34, −20)	408	7.241	0.186	0.00000
**rLPC seed**						
Middle frontal gyrus	BA 8	(26, 30, 52)	17027	14.827	0.480	0.00000
Superior temporal gyrus	BA 39	(52, −60, 26)	7214	24.514	0.901	0.00000
Cuneus	BA 18	(2, −70, 30)	7204	17.547	0.501	0.00000
Middle temporal gyrus	BA 39	(−44, −68, 26)	4980	20.052	0.566	0.00000
Middle temporal gyrus	BA 20	(−54, −8, −22)	2654	9.443	0.267	0.00000
Insular cortex	BA 13	(42, 6, −4)	694	−7.820	−0.210	0.00000
Fusiform gyrus	BA 37	(−30, −36, −16)	358	7.388	0.271	0.00000
Parahippocampal gyrus	BA 36	(30, −20, −28)	316	5.600	0.147	0.00000
Insular cortex	BA 48	(−36, 14, 8)	197	−6.373	−0.135	0.00000
**pACC seed**						
Anterior cingulate cortex	BA 32	(−2, 38, 16)	25640	46.025	2.517	0.00000
Inferior temporal gyrus	BA 20	(−60, −56, −16)	2289	−8.354	0.160	0.00000
Inferior parietal lobule	BA 48	(−44, −34, 32)	2285	−2.731	−0.079	0.00000
Precuneus	BA 7	(8, −60, 70)	2062	−9.274	−0.176	0.00000
Middle occipital gyrus	BA 37	(50, −64, −10)	1555	−6.903	−0.132	0.00000
Inferior parietal cortex	BA 18	(58, −50, 50)	365	6.027	0.150	0.00000
**PCC/PC seed**						
Middle frontal gyrus	BA 8	(26, 40, 44)	12514	12.037	0.453	0.00000
Precuneus	BA 23	(2, −62, 26)	8240	51.265	2.433	0.00000
Angular gyrus	BA 39	(−44, −62, 26)	2753	12.495	0.521	0.00000
Middle temporal gyrus	BA 21	(−66, −28, −8)	2310	8.849	0.229	0.00000
Angular gyrus	BA 39	(54, −62, 34)	2107	13.294	0.493	0.00000
Inferior temporal gyrus	BA 20	(56, −4, −38)	1751	11.132	0.212	0.00000
Insular cortex	BA 48	(48, 12, 4)	1197	−7.101	−0.226	0.00000
Supramarginal gyrus	BA 2	(54, −34, 38)	1075	−7.354	−0.264	0.00000
Insular cortex	BA 48	(−36, 2, −4)	905	−7.141	−0.165	0.00000
Middle frontal gyrus	BA 46	(−40, 54, 8)	837	−8.354	−0.215	0.00000
Inferior frontal gyrus	BA 45	(44, 40, 2)	712	−6.300	−0.239	0.00000
Middle occipital gyrus	BA 18	(−30, −90, 8)	486	−7.160	−0.167	0.00000
Fusiform gyrus	BA 37	(−30, −36, −16)	342	7.493	0.204	0.00000
Parahippocampal gyrus	BA 35	(26, −22, −24)	229	9.158	0.199	0.00000
Superior temporal gyrus	BA 38	(40, 20, −34)	172	5.631	0.157	0.00006
Middle occipital gyrus	BA 37	(−50, −62, −10)	124	−6.009	−0.159	0.00010
**Ig2 seed**						
Insular cortex	BA 13	(42, −14, −8)	9980	63.964	0.669	0.00000
Postcentral gyrus	BA 40	(−58, −26, 16)	9729	16.404	0.298	0.00000
Anterior cingulate cortex	BA 24	(4, 22, 24)	6178	11.215	0.209	0.00000
Cuneus	BA 18	(−12, −72, 6)	5566	10.453	0.163	0.00000
Middle frontal gyrus	BA 46	(−32, 44, 22)	329	7.780	0.154	0.00000
Middle frontal gyrus	BA 9	(38, 26, 54)	184	−5.517	−0.085	0.00000

*We report clusters having significant connections from the seed region, cluster size, and the peak-voxel location in each cluster.*

**TABLE 2:** Statistical significance of the group-level functional connectivity generated during resting-state condition.

Connectivity (Resting state)	Brodmann area	MNI (x,y,z)	*Size*	*Peak-T*	*Peak-beta*	*Size p-FDR*
**PCC seed**						
Precuneus	BA 7	(−2, −64, 40)	22585	33.057	0.930	0.00000
Medial frontal gyrus	BA 11	(8, 54, −12)	8607	11.036	0.303	0.00000
Middle frontal gyrus	BA 9	(−28, 42, 42)	2296	9.627	0.263	0.00000
Insular cortex	BA 22	(50, 2, −2)	1748	−7.633	−0.161	0.00000
Middle temporal gyrus	BA 21	(52, 0, −26)	613	7.965	0.195	0.00000
Middle temporal gyrus	BA 21	(−62 0 −26)	311	6.144	0.133	0.00000
**mPFC seed**						
Medial orbital gyrus	BA 11	(0, 50, −10)	23406	27.766	1.122	0.00000
Posterior cingulate cortex	BA 23	(−10, −54, 22)	6945	16.959	0.422	0.00000
Supramarginal gyrus	BA 40	(44, −34, 38)	4216	−9.262	−0.150	0.00000
Inferior parietal lobe	BA 40	(−38, −42, 44)	4023	−8.222	−0.270	0.00000
Angular gyrus	BA 39	(−46, −64, 30)	2171	13.206	0.383	0.00000
Angular gyrus	BA 39	(52, −68, 34)	1983	12.751	0.433	0.00000
Inferior temporal gyrus	BA 37	(−58, −60, −8)	877	−7.902	−0.191	0.00000
Fusiform gyrus	BA 37	(54, −50, −24)	812	−9.106	−0.167	0.00000
Parahippocampal gyrus	BA 30	(24, −20, −24)	571	7.417	0.230	0.00000
Inferior frontal gyrus	BA 44	(−48, 8, 20)	422	−5.921	−0.175	0.00000
Middle occipital gyrus	BA 18	(34, −92, 10)	384	9.068	0.194	0.00000
**lLPC seed**						
Superior frontal gyrus	BA 8	(−30, 24, 58)	27546	14.534	0.440	0.00000
Angular gyrus	BA 19	(−40, −74, 38)	12347	28.357	0.992	0.00000
Middle temporal gyrus	BA 39	(40, −66, 28)	4118	18.926	0.446	0.00000
Middle temporal gyrus	BA 20	(−60, −44, −14)	1331	12.042	0.366	0.00000
Superior temporal gyrus	BA 38	(−52, 2, −4)	788	−6.582	−0.190	0.00000
Parahippocampal gyrus	BA 36	(26, −28, −20)	615	8.237	0.154	0.00000
Fusiform gyrus	BA 37	(−28, −38, −18)	551	11.315	0.353	0.00000
Middle cingulate cortex	BA 32	(−8, 16, 36)	355	−5.982	−0.151	0.00000
Supramarginal gyrus	BA 40	(−52, −26, 14)	180	−5.442	−0.158	0.00000
Cuneus	BA 18	(22, −88, 8)	161	−5.987	−0.166	0.00001
**rLPC seed**						
Middle frontal gyrus	BA 8	(28, 32, 52)	17537	17.430	0.542	0.00000
Superior temporal gyrus	BA 39	(48, −58, 22)	10591	25.784	0.839	0.00000
Middle temporal gyrus	BA 39	(−42, −64, 24)	4687	21.402	0.546	0.00000
Middle temporal gyrus	BA 20	(−60, −44, −14)	2905	10.013	0.275	0.00000
Middle temporal gyrus	BA 21	(52, −4, −26)	2185	9.641	0.289	0.00000
Insular cortex	BA 13	(40, 4, −2)	1521	−6.337	−0.207	0.00000
Parahippocampal gyrus	BA 30	(26, −20, −24)	653	7.891	0.215	0.00000
Fusiform gyrus	BA 37	(−28, −38, −16)	652	8.397	0.235	0.00000
Middle cingulate cortex	BA 24	(2, 16, 40)	342	−6.416	−0.188	0.00000
Cuneus	BA 19	(22, −82, 18)	193	−5.855	−0.159	0.00000
Lingual gyrus	BA 18	(−10, −64, −6)	160	−5.583	−0.132	0.00002
**pACC seed**						
Anterior cingulate cortex	BA 32	(−2, 38, 16)	24791	53.786	2.502	0.00000
Inferior parietal cortex	BA 7	(34, −50, 58)	1299	−7.197	−0.170	0.00000
Fusiform gyrus	BA 20	(54, −36, −26)	368	−9.512	−0.128	0.00000
Paracentral lobule	BA 4	(−14, −38, 64)	143	−4.862	−0.105	0.00005
**PCC/PC seed**						
Superior frontal gyrus	BA 10	(−4, 64, −6)	11827	14.727	0.388	0.00000
Precuneus	BA 23	(2, −62, 26)	7292	49.964	2.393	0.00000
Insular cortex	BA 48	(34, 16, 6)	3232	−13.322	−0.252	0.00000
Middle temporal gyrus	BA 38	(−42, 14, −32)	2957	8.952	0.193	0.00000
Middle temporal gyrus	BA 39	(−48, −66, 28)	2802	13.988	0.515	0.00000
Supramarginal gyrus	BA 2	(66, −24, 28)	2555	−11.081	−0.281	0.00000
Superior temporal gyrus	BA 39	(56, −60, 28)	2461	15.897	0.506	0.00000
Middle temporal gyrus	BA 21	(54, −2, −26)	2302	9.784	0.345	0.00000
Middle cingulate cortex	BA 32	(6, 14, 42)	996	−8.940	−0.169	0.00000
Middle frontal gyrus	BA 46	(−32, 46, 28)	790	−7.645	−0.208	0.00000
Parahippocampal gyrus	BA 36	(28, −16, −30)	743	8.167	0.155	0.00000
Precuneus	BA 7	(−12, −58, 60)	388	−5.490	−0.142	0.00000
**Ig2 seed**						
Insular cortex	BA 13	(42, −12, −8)	13955	59.166	1.709	0.00000
Middle cingulate cortex	BA 31	(6, −52, 32)	6103	−6.661	−0.146	0.00000
Parahippocampal gyrus	BA 30	(−20, −42, −8)	464	5.728	0.108	0.00000
Cuneus	BA 18	(16, −72, 8)	157	6.563	0.129	0.00003
Middle frontal gyrus	BA 10	(4, 68, 18)	121	−6.164	−0.081	0.00005

*We report clusters having significant connections from the seed region, cluster size, and the peak-voxel location in each cluster.*

**FIGURE 2:**
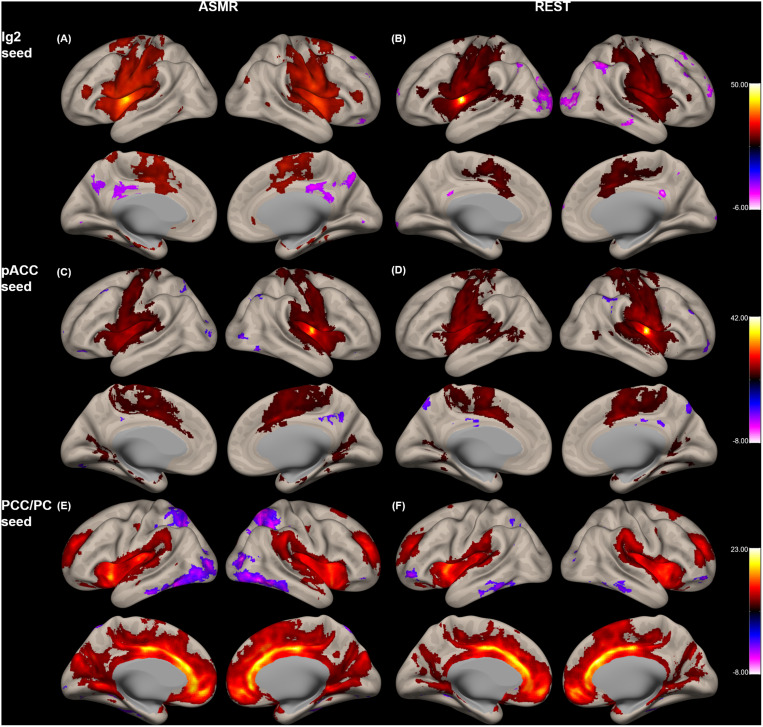
Group-level functional connectivity of the *t*-statistic in the other networks during resting-state, and in response to ASMR effects. Functional connectivity strengths in terms of *t*-statistics were thresholded at a significance level of false discovery rate (FDR)-corrected *p* < 0.05, and overlaid on a cortical surface atlas. Functional connectivity of the right posterior insular cortex seed (Ig2) region in response to ASMR **(A)**, and in resting-state **(B)**. Functional connectivity of the pregenual anterior cingulate cortex (pACC) seed region in response to ASMR **(C)**, and in resting-state **(D)**. Functional connectivity of the posterior cingulate cortex/precuneus (PCC/PC) seed regions in response to ASMR **(E)**, and in resting state **(F)**.

Figure 2 shows the group-level functional connectivity of the *t*-statistic in the affective touch, self-, and other-networks generated during either ASMR or resting-state conditions. For the affective touch network with Ig2 seed region, the significant clusters were estimated in the insular cortex and postcentral gyrus in both conditions of resting-state and ASMR. In the self-network with the pACC seed region, we found the positive functional connectivity of the anterior cingulate cortex. In other-network with the PCC/PC seed region, the positive functional connectivity was observed in the angular gyrus, precuneus, and frontal regions extending orbitofrontal and medial prefrontal cortices.

**FIGURE 3:**
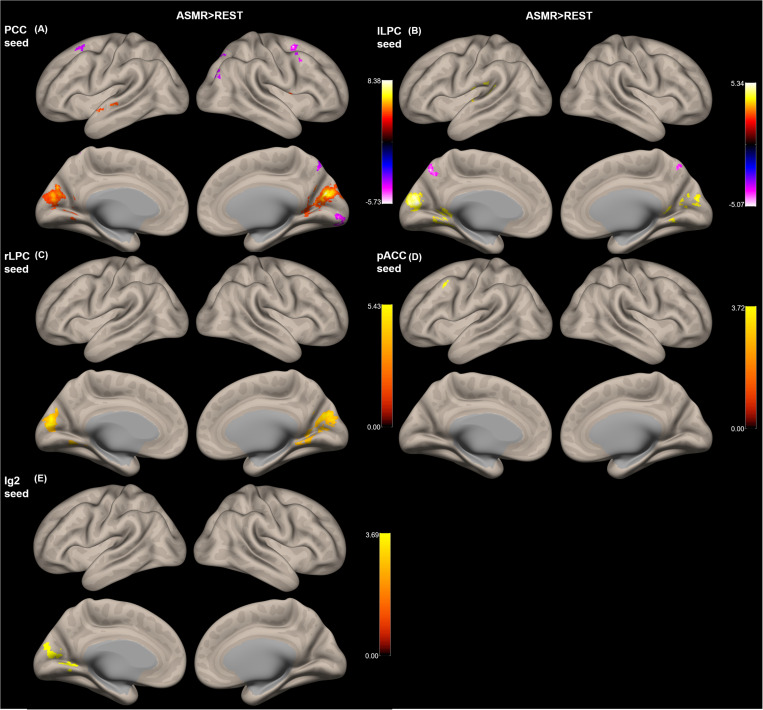
Group-level functional connectivity of the *t*-statistic obtained by the “ASMR > resting-state” contrast. The default mode networks with seed regions of **(A)** the posterior cingulate cortex (PCC), **(B)** left lateral parietal cortex (lLPC), and **(C)** right lateral parietal cortex (rLPC). **(D)** The self-network with the pregenual anterior cingulate cortex (pACC) seed region. **(E)** Affective touch network with the posterior insular cortex (Ig2) seed region. There were no significant clusters in the default mode network with the mPFC seed region and the other-network with the PCC/PC seed region.

Figure 3 shows the group-level functional connectivity of the t-statistic obtained by the “ASMR > resting-state” contrast. Table 3 summarizes statistical significance of clusters functionally connected to the seed regions of the PCC, l/rLPC, pACC, and Ig2, and their peak coordinates. There were no significant clusters in the DMN with the mPFC seed region and the other-network with the PCC/PC seed region. In the DMN with the PCC seed region, 5 clusters having positive functional connectivity were significantly detected in peaks in the cuneus, superior/middle temporal gyri, and lingual gyrus. In addition, 6 clusters having negative functional connectivity were significantly detected in peaks in the superior/middle frontal gyri, middle occipital lobe, precuneus, and visual area. In the DMN with the lLPC seed region, 2 positive and 1 negative clusters were observed in peaks in the superior temporal gyrus and visual area (calcarine sulcus), and precuneus, respectively. In the DMN with the rLPC seed region, 2 positive clusters were generated in peaks in the cuneus and lingual gyrus. In the self-network with the pACC seed region, a positive cluster was detected in peaks in the middle frontal lobe. In the affective touch network with the the Ig2 seed region, one cluster having positive functional connectivity was observed in peaks in the cuneus.

**TABLE 3:** Statistical significance of the group-level functional connectivity obtained by the “ASMR > resting-state” contrast.

Connectivity (ASMR > REST)	Brodmann area	MNI (x,y,z)	*Size*	*Peak-t*	*Peak-beta*	*Peak p-unc*	*Size p-FDR*
**PCC seed**							
Cuneus	BA 18	(8, −74, 22)	1451	8.799	0.283	0.00000	0.00000
Superior frontal gyrus	BA 6	(24, 4, 56)	176	−5.498	−0.159	0.00000	0.00001
Visual area	BA 18	(10, −90, −6)	173	−6.020	−0.207	0.00000	0.00001
Lingual gyrus	BA 18	(−18, −70, 2)	59	4.290	0.176	0.00021	0.01692
Precuneus	BA 7	(6, −66, 48)	49	−4.552	−0.171	0.00010	0.02626
Superior temporal gyrus	BA 48	(54, 0, 0)	49	4.840	0.178	0.00004	0.02626
Superior temporal gyrus	BA 22	(−54, −2, −8)	42	5.822	0.158	0.00000	0.04210
Precuneus	BA 7	(−6, −64, 66)	37	−4.331	−0.219	0.00018	0.04281
Middle frontal gyrus	BA 8	(−24, 16, 58)	37	−4.434	−0.177	0.00014	0.04281
Middle occipital lobe	BA 39	(40, −78, 24)	37	−4.187	−0.200	0.00027	0.04281
Middle temporal gyrus	BA 21	(−62, −20, −6)	37	5.742	0.140	0.00000	0.04281
**lLPC seed**							
Visual area	BA 17	(−6, −78, 16)	526	5.702	0.186	0.00000	0.00000
Superior temporal gyrus	BA 22	(−56, −32, 10)	266	5.599	0.168	0.00000	0.00000
Precuneus	BA 7	(−6, −66, 50)	118	−5.131	−0.176	0.00002	0.00296
**rLPC seed**							
Cuneus	BA 18	(8, −76, 22)	1014	5.812	0.211	0.00000	0.00000
Lingual gyrus	BA 18	(−14, −64, −6)	113	5.501	0.187	0.00000	0.00002
**pACC seed**							
Middle frontal lobe	BA 9	(−50, 18, 44)	53	4.426	0.183	0.00014	0.03391
**Ig2 seed**							
Cuneus	BA 17	(−10, −68, 6)	301	5.565	0.143	0.00000	0.00000

*We report clusters having significant connections from the seed region, the peak-voxel location in each cluster, and the corresponding *t*-, *beta*-, and *p*-values.*

The beta-values of the group-level functional connectivity for ASMR, resting-state, and ASMR > resting-state contrast are provided in Supplementary Figures.

### Behavioral Data

**FIGURE 4:**
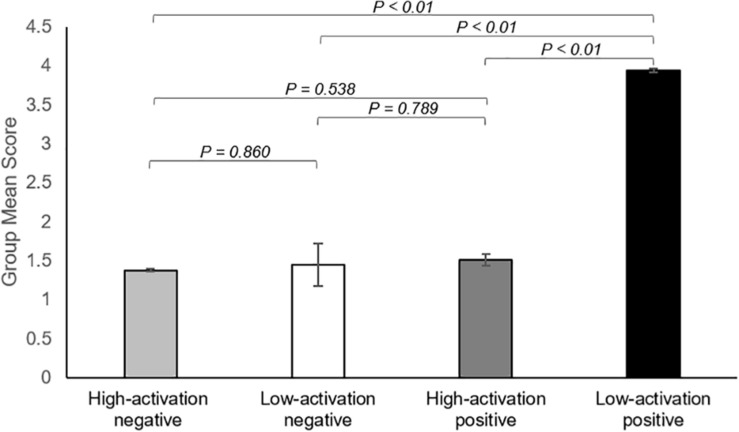
Summary of the results showing changes in affect state after viewing ASMR, relative to before watching ASMR. Bar graphs represent group mean scores for affective state assessed using the Multi-Affect Indicator (Warr, 1990). All variables range from 1 to 7. For self-reported changes in affect, 1 = much less; 7 = much more. The participants had the most increase in low-activation positive state during the ASMR condition among four affective states: low-activation positive state (group mean ± standard deviation: 3.94 ± 1.46), high-activation positive state (1.51 ± 0.63), low-activation negative state (1.45 ± 0.64), and high-activation negative state (1.38 ± 0.78). Statistical significance was determined by a *p*-value of less than 0.05.

There was a significant overall main effect on the affective response while watching ASMR video clips. As shown in Figure 4, participants had the most increase in low-activation positive state during the ASMR condition among four affective states that we have considered: LAP (group mean ± standard deviation: 3.94 ± 1.46), HAP (1.51 ± 0.63), LAN (1.45 ± 0.64), and HAN (1.38 ± 0.78). Statistical significance of the comparison between two selected states are as follows: LAP > HAP [beta = 2.429, *t* = 8.349, *p* = 5.86 × 10^–9^, df = 27, 95% confidence interval of the mean = (1.832–3.025); LAP > LAN (beta = 2.488, *t* = 8.471, *p* = 4.39 × 10^–9^, df = 27, 95% confidence interval of the mean = (1.885–3.091); LAP > HAN (beta = 2.560, *t* = 7.638, *p* = 3.25 × 10^–8^, df = 27, 95% confidence interval of the mean = (1.872–3.247)]. Table 4 summarizes the statistical significance of affective states in response to ASMR.

**TABLE 4:** Mean and standard deviation of behavioral score among emotional states.

Item	Average score		Standard deviation		
Nervous	1.464		0.865		
Anxious	1.321		0.847		
Tense	1.357		0.934		
**HAN**	**1.381**		**0.775**		
Depressed	1.214		0.619		
Dejected	2.107		1.496		
Hopeless	1.036		0.186		
**LAN**	**1.452**		**0.644**		
Enthusiastic	1.536		0.906		
Joyful	1.786		1.013		
Excited	1.214		0.674		
**HAP**	**1.512**		**0.627**		
Calm	3.964		1.742		
Relaxed	4.071		1.731		
At ease	3.786		1.820		
**LAP**	**3.940**		**1.456**		

**Paired *t*-test**	***p***	***t***	**beta**	**(95% CI)**	***df***

LAP-HAP	0.00000	8.349	2.429	(1.832–3.025)	27
LAP-LAN	0.00000	8.471	2.488	(1.885–3.091)	27
LAP-HAN	0.00000	7.638	2.560	(1.872–3.247)	27
HAP-LAN	0.6858	0.409	0.060	(-0.239–0.358)	27
HAP-HAN	0.4957	0.691	0.131	(-0.258–0.520)	27
LAN-HAN	0.6078	0.519	0.071	(-0.211–0.354)	27

*We report *t*-test results for comparing affective states during ASMR. HAN, High-activation negative state; LAN, Low-activation negative state; HAP, High-activation positive state; LAP, Low-activation positive state; df, Degrees of freedom.*

Correlation coefficients between each of the four affective states and ASMR condition-specific connectivity changes are summarized in Table 5. In the DMN with the PCC seed region, significantly negative correlation was estimated between HAN and clusters with peaks in the lingual gyrus. Associations of HAP with clusters of the cuneus and lingual gyrus were also negatively correlated. In the affective touch and self-/other-networks, there were no significant correlation between the affective state scores and the ASMR-condition specific connectivity changes.

**TABLE 5:** Statistical results of correlation coefficients between each of the four affective states and ASMR condition-specific connectivity changes.

Connectivity-behavioral correlation	MNI (x,y,z)	*r*	*p*
**PCC seed**			
HAN-Lingual gyrus *	(−18, −70, 2)	−0.411	0.030
**rLPC seed**			
HAP−Cuneus **	(8, −76, 22)	−0.5085	0.006
HAP−Lingual gyrus**	(−14, −64, −6)	−0.497	0.007

**p–value < 0.01, *p–value < 0.05. PCC, Posterior cingulate cortex; rLPC, Right lateral parietal cortex. HAN, High-activation negative state; HAP, High-activation positive state.

## Discussion

In this study, we sought to test whether changes in functional connectivity within specific networks, including the DMN, affective touch network, and self-/other-networks occurred during ASMR. As a result, relative to connectivity in the resting-state, significantly altered connectivity of seed regions during viewing of ASMR-eliciting stimulus was found in the main hub composing each network. Furthermore, we confirmed that the strength of connectivity in involved in visual information processing was negatively correlated with the behavior score, including the HAN, and HAP states. We now discuss the implications of these results in more detail.

### Default Mode Network (ASMR > REST)

Our results showed that in the DMN, functional connectivity between the PCC seed region and the superior/middle temporal gyri, cuneus, and lingual gyrus were significantly increased during ASMR condition, compared to the resting-state. Previous functional imaging studies (Carrington and Bailey, 2009; Spreng et al., 2009) have found that the PCC and superior temporal gyrus (STG) are involved in the “mentalizing,” also known as “theory of mind” that is an ability to make inferences about other people’s mental states [i.e., an understanding that the behaviors of others is determined by their desires, attitude, and beliefs (Frith and Frith, 2003)]. Specifically, Castelli et al. (2000) revealed that the superior temporal region was activated while watching silent or computer-presented animations, and this process was related to the attribution of mental states. Fletcher et al. (1995) reported significantly increased cerebral blood flow in the PCC during the condition necessitating the attribution of mental task. Therefore, the increased functional connectivity between the STG and PCC during ASMR condition can be associated with the increased covariance of the STG and the PCC activities compared to the resting-state, which may be interpreted as activation of mentalizing process to infer others’ mental and emotional states by observing objects and perceiving intended actions and using ourselves to simulate their experience to understand them (Blakemore and Decety, 2001; Allen et al., 2003; Frith and Frith, 2006; Liew et al., 2011; Riekki et al., 2018).

We also found the reduced connectivity between the dorsolateral prefrontal cortex (dlPFC) and the PCC during ASMR condition, compared with the resting-state. Lévesque et al. (2003) reported that the dlPFC was involved in inhibition processing such as voluntary suppression of a negative emotion (sadness) while the participants suppressed their emotional reaction to the sad stimuli. For the PCC, this region has been known to be a part of network for emotion evaluation (Lee and Siegle, 2012), including an automatic perception for the emotion salience of stimulus (Maddock, 1999). Thus, compared to the resting state, the decreased functional connectivity between the dlPFC and PCC during ASMR condition can be interpreted as the decrease in voluntary suppression of negative emotion. This process may occur due to the nature of ASMR triggers that often lead to response of psychologically pleasant effects (Poerio et al., 2018).

With the DMN of the bilateral LPC seed regions, we found that the functional connectivity between the l/rLPC seeds and the visual areas of the cuneus and calcarine sulcus was significantly higher during the ASMR condition than during the resting-state. The cuneus is involved in visual information processing that interacts with the primary visual cortex (Vanni et al., 2001) and is known to integrate somatosensory information with other sensory stimuli (Price, 2000). In addition, the LPC is involved in receiving a visual input from the occipital regions, which belong to the dorsal stream of visual processing (Rizzolatti and Matelli, 2003). In terms of the visual stimuli, in our experiment, ASMR-eliciting video clips were much richer in visual information than the instruction for resting-state condition (with eyes fixated on a cross). Therefore, greater functional connectivity of the cuneus and calcarine sulcus within the DMN may reflect the increased visual input and processing from ASMR-eliciting stimuli through functional connectivity, compared to the resting-state condition.

### Affective Touch and Self-Networks (ASMR > REST)

This study showed significant connectivity differences not only in the DMN but also in other network areas, including affective touch network, and self-network. In terms of the affective touch network, we found a greater connectivity between the Ig2 and the cuneus of the occipital region during the ASMR condition than the resting-state. The cuneus is a part of the visual areas and engages in processing of visual input (Waberski et al., 2008) and the insular cortex integrates information from multiple modalities, including visual and auditory sensory modalities (Bamiou et al., 2003). Thus, the increased connection between Ig2 and cuneus indicates the higher visuoauditory influence of ASMR stimulus.

In terms of the self-network involved in the reflection of one’s own experiences against other stimuli (Northoff et al., 2006), we found an increased connectivity between the pACC and the mPFC during ASMR condition, compared to resting-state. Murray et al. (2012) revealed that the mPFC and dorsal anterior cingulate cortex were activated in the self-referencing processing state rather than the other-relevant processing, and Gusnard et al. (2001) showed that these regions were particularly involved in self-referential processing in emotion domain. In addition, Northoff et al. (2006) reported that cortical midline structures including the mPFC and pACC mediate self-referential processing in psychological or physical domain such as autobiographical, emotional, and motor stimuli. Therefore, the increased connectivity between the pACC and the mPFC during ASMR may reflect the self-referential processing triggered by ASMR stimulus.

### Correlation Between Connectivity and Affective State

Although the major focus of this study is the connectivity on which the effects of ASMR are neural underpinnings, a correlation analysis was performed to investigate how these changed connections relate to the feelings felt during ASMR. As a result, in the PCC region, significantly negative correlation was estimated between clusters with peaks in the lingual gyrus and HAN. For rLPC seed region, connectivities in clusters of the lingual gyrus and cuneus were also negatively correlated in HAP. The PCC receives visual information from visual systems (Vogt et al., 2006) and the LPC also accepts visual input through dorsal stream (Rizzolatti and Matelli, 2003). The ASMR stimulus contains audio-visual stimuli that lead to a positive emotional response to calmness and a tingling sensation that emerges from a positive emotion (Barratt and Davis, 2015). Thus, these results imply that visual information processing in response to high arousal states can be weakened by ASMR-eliciting stimuli.

As a limitation of this finding, we did not explicitly measure the affective outcomes of resting state using the behavioral questionnaire [e.g., the Multi-Affect Indicator (Warr, 1990; Warr et al., 2014)]. As described in the Behavioral Data Analysis section, the participants were instructed to indicate how they felt while watching the ASMR video clip during the MRI scan, compared to before they watched the video. Therefore, individual behavioral scores that we measured may reflect relative affective states of ASMR condition to resting state. However, a control acquisition of the behavioral questionnaire after the resting state session would be required to compare the affective state changes between resting-state and ASMR conditions more explicitly. Thus, caution should be exercized when interpreting the correlation coefficient between functional connectivity estimates and behavioral scores used in this study.

In conclusion, using fMRI functional connectivity estimates, we explored the ASMR-condition specific connectivity changes in the DMN, self-/other-networks, and the affective touch network. Compared with the resting-state functional connectivity, we found that several connections within the selected networks were significantly altered while watching ASMR video. In particular, the connections between the PCC and the superior temporal gyrus, between the pACC and the mPFC, and between the Ig2 and the cuneus were significantly greater during ASMR condition than resting state. These results suggest that ASMR process can be associated with ongoing interaction between regional activity that are involved in the integration of visual and auditory information followed by the mentalizing and self-referential processing. In terms of the relationship between connectivity and affective state changes, we found that ASMR-induced affective states (i.e., high activation negative and high activation positive state) were significantly negatively correlated with functional connectivity involved in visual information processing. These results imply that high arousal states can be attenuated in the process of perception of ASMR-eliciting stimuli. Our findings have implications for neurophysiological mechanisms of an ASMR effects in relation to functional connectivity changes.

## Data Availability Statement

The fMRI data that support the findings of this study are available from the corresponding author on request.

## Ethics Statement

The studies involving human participants were reviewed and approved by the Institutional Review Board of Korea Basic Science Institute. The patients/participants provided their written informed consent to participate in this study.

## Author Contributions

SL designed the study. SL and JK conducted the experiment and performed the fMRI data acquisition. SL and ST performed the data analysis, discussed the study idea, analysis, and results, and wrote the manuscript. All authors reviewed the manuscript.

## Conflict of Interest

The authors declare that the research was conducted in the absence of any commercial or financial relationships that could be construed as a potential conflict of interest.

## Supplementary Material

FIGURE S1 Group-level functional connectivity of the beta-value in the default mode network during resting-state, and in response to ASMR effects. Functional connectivity of the posterior cingulate cortex seed region in response to **(A)** ASMR, and **(B)** resting-state. Functional connectivity of the medial prefrontal cortex seed region in response to **(C)** ASMR, and **(D)** resting-state. Functional connectivity of the left lateral parietal cortex seed region in response to **(E)** ASMR, and **(F)** resting-state. Functional connectivity of the right lateral parietal cortex seed region in response to **(G)** ASMR, and **(H)** resting-state.

FIGURE S2 Group-level functional connectivity of the beta-value in the affective touch, self-, and other-networks during resting-state, and in response to ASMR effects. Functional connectivity of the right posterior insular cortex seed region in response to **(A)** ASMR, and **(B)** resting-state. Functional connectivity of the pregenual anterior cingulate cortex seed region **(C)** in response to ASMR, and **(D)** resting-state. Functional connectivity of the posterior cingulate cortex/precuneus seed region in response to **(E)** ASMR, and **(F)** resting-state.

FIGURE S3 Group-level functional connectivity of the beta-value for ASMR > resting-state contrast. Default mode networks with seed regions of **(A)** the posterior cingulate cortex, **(B)** medial prefrontal cortex, **(C)** left lateral parietal cortex, and **(D)** right lateral parietal cortex. **(E)** Self-network with the pregenual anterior cingulate cortex seed region. **(F)** Other-network with the posterior cingulate cortex/precuneus seed region. **(G)** Affective touch network with the posterior insular cortex seed region.
