# Supplementary figures and images for: Effects of Autonomous Sensory Meridian Response on the Functional Connectivity as Measured by Functional Magnetic Resonance Imaging
Source: Front Behav Neurosci. 2020 Aug 27;14:154. doi: 10.3389/fnbeh.2020.00154 (PMC7481390; doi:10.3389/fnbeh.2020.00154)

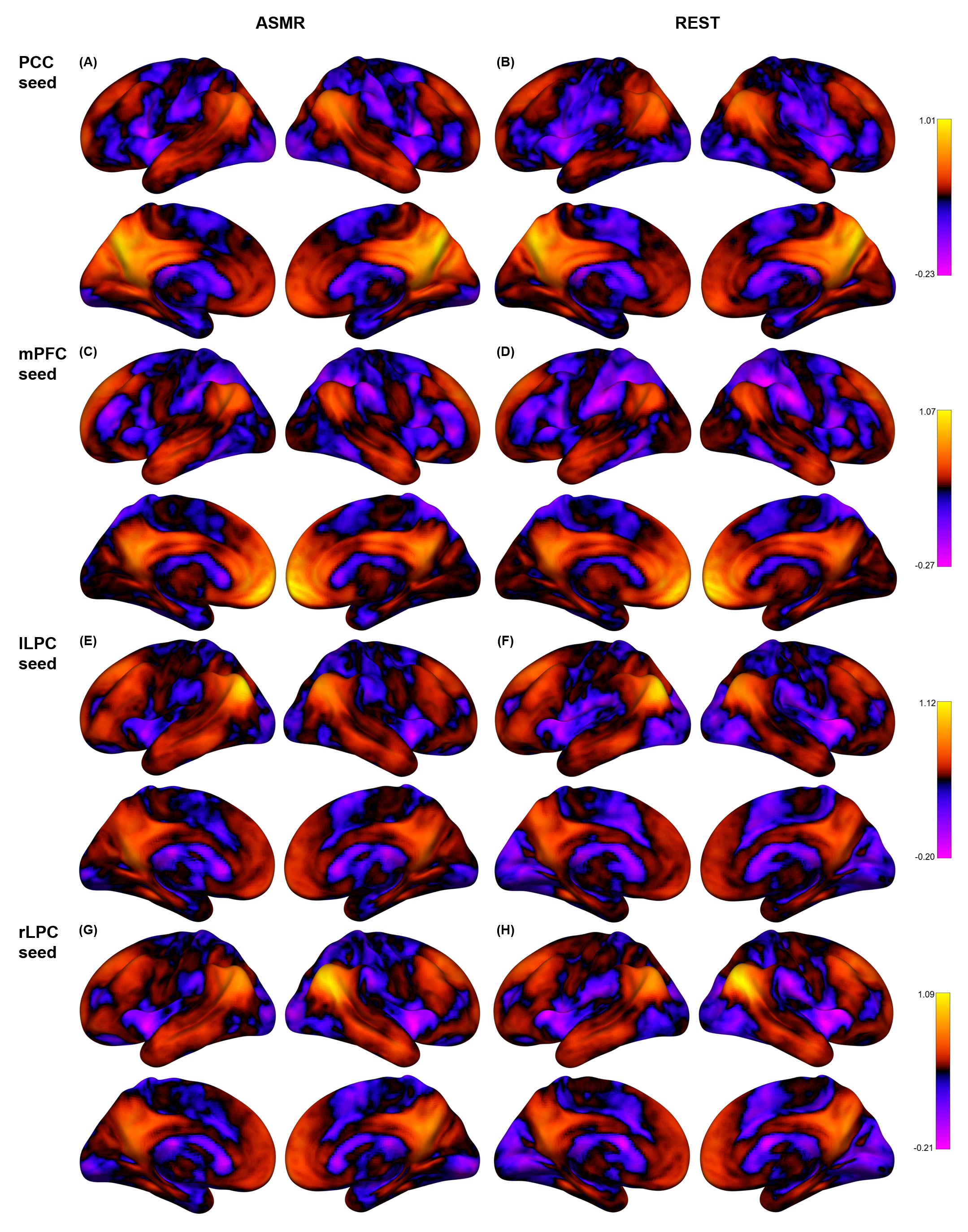

Supplement: FIGURE S1 — Group-level functional connectivity of the beta-value in the default mode network during resting-state, and in response to ASMR effects. Functional connectivity of the posterior cingulate cortex seed region in response to (A) ASMR, and (B) resting-state. Functional connectivity of the medial prefrontal cortex seed region in response to (C) ASMR, and (D) resting-state. Functional connectivity of the left lateral parietal cortex seed region in response to (E) ASMR, and (F) resting-state. Functional connectivity of the right lateral parietal cortex seed region in response to (G) ASMR, and (H) resting-state. [file Image_1.TIF]

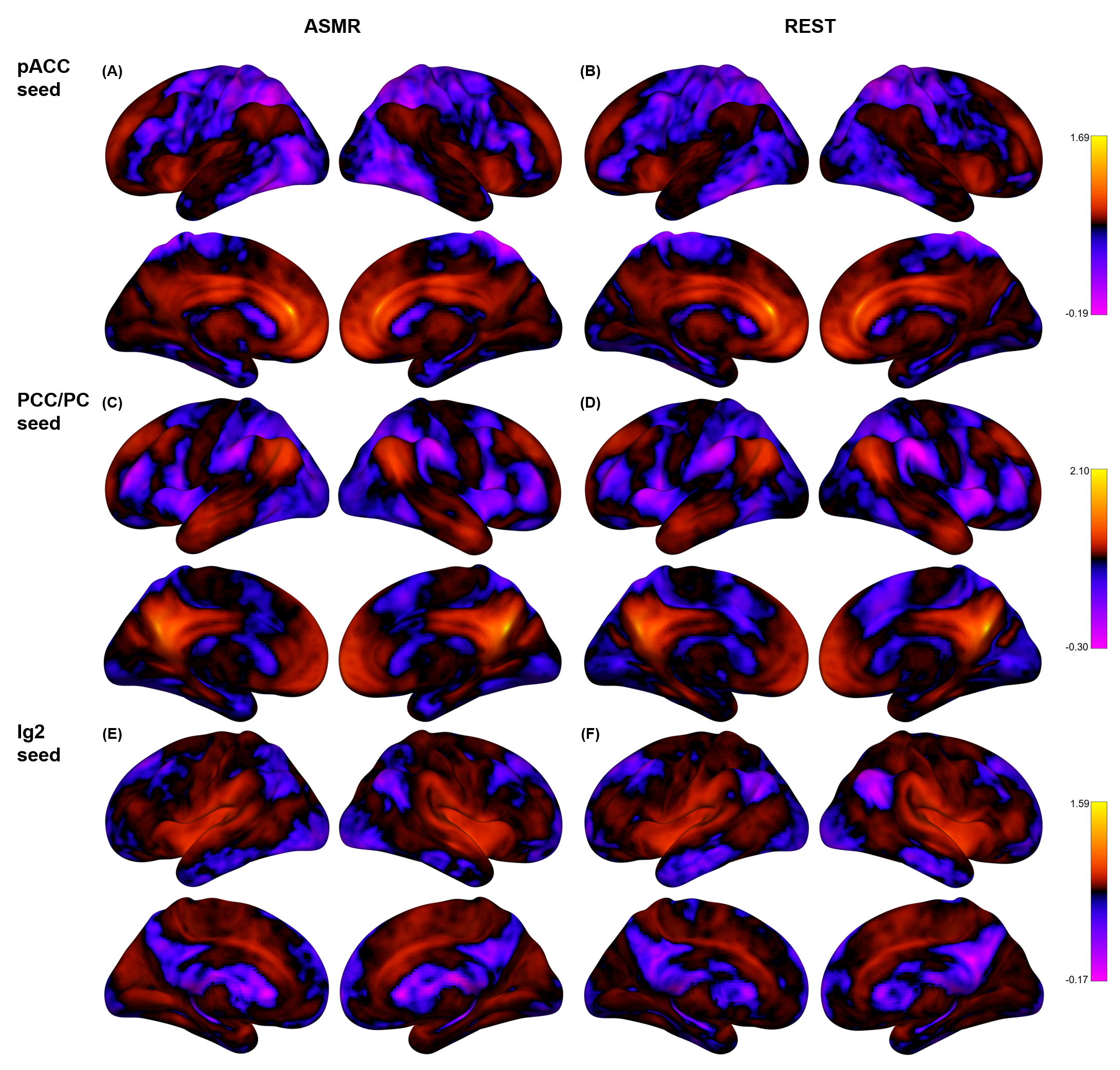

Supplement: FIGURE S2 — Group-level functional connectivity of the beta-value in the affective touch, self-, and other-networks during resting-state, and in response to ASMR effects. Functional connectivity of the right posterior insular cortex seed region in response to (A) ASMR, and (B) resting-state. Functional connectivity of the pregenual anterior cingulate cortex seed region (C) in response to ASMR, and (D) resting-state. Functional connectivity of the posterior cingulate cortex/precuneus seed region in response to (E) ASMR, and (F) resting-state. [file Image_2.TIF]

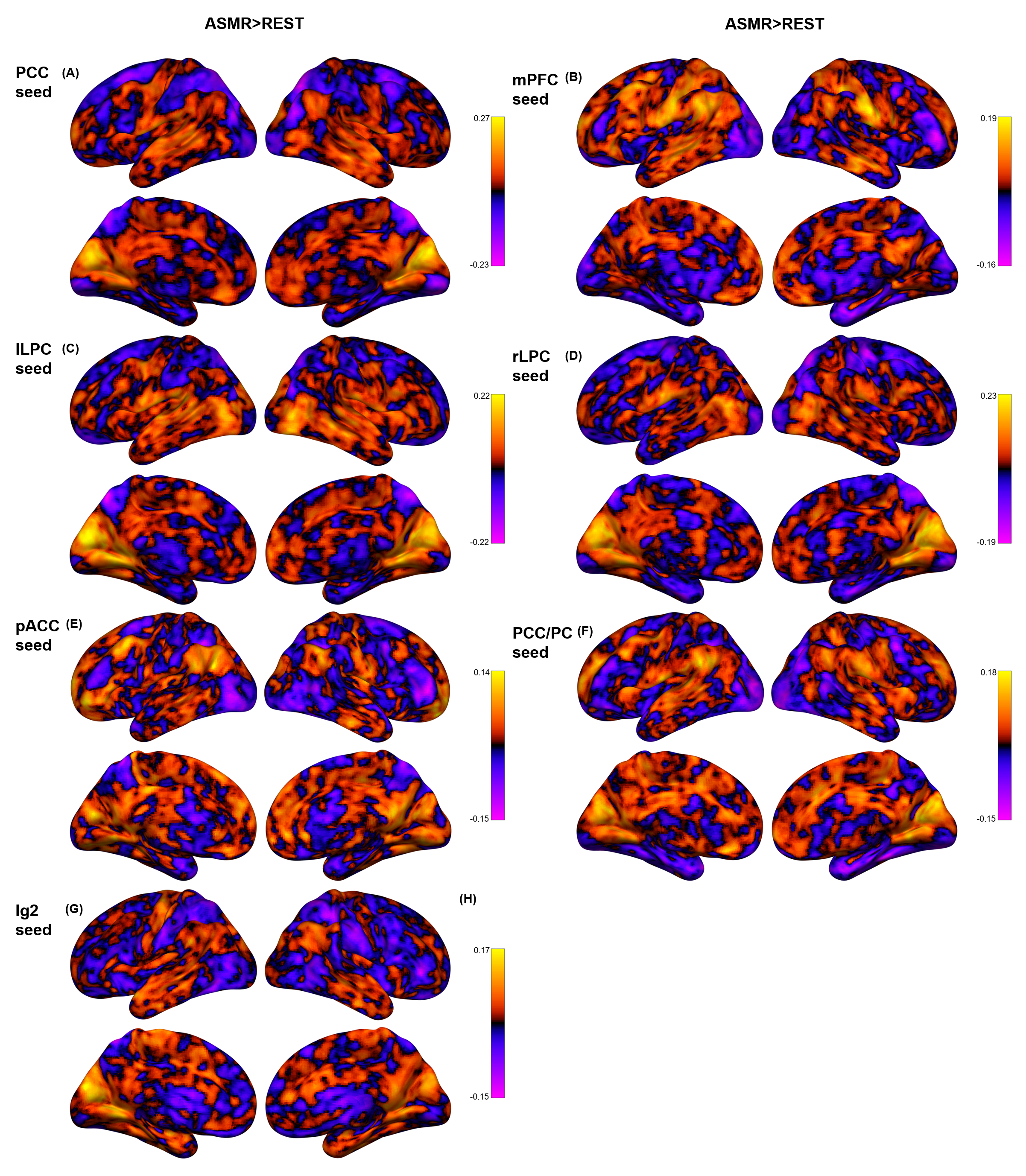

Supplement: FIGURE S3 — Group-level functional connectivity of the beta-value for ASMR > resting-state contrast. Default mode networks with seed regions of (A) the posterior cingulate cortex, (B) medial prefrontal cortex, (C) left lateral parietal cortex, and (D) right lateral parietal cortex. (E) Self-network with the pregenual anterior cingulate cortex seed region. (F) Other-network with the posterior cingulate cortex/precuneus seed region. (G) Affective touch network with the posterior insular cortex seed region. [file Image_3.TIF]
